# Functional Tests of the Kidney in Uremia

**Published:** 1914-02

**Authors:** 


FUNCTIONAL TESTS OF THE KIDNEY IN UREMIA. N. B. Foster, New York, *Arch. Internal Med.*, October 15, 1913, calls attention to certain limitations of laboratory aids, and to exceptions which many cases present to what is perhaps a doubtful average. The cause of uremia is not known, but the common conception relates the condition to nephritis. At necropsy marked renal degeneration is requisite for confirmation of the diagnosis, and with this is usually found edema of the brain and often colitis. The value of a test having for its aim the estimation of renal function can be measured by the results elicited in demonstrated cases of uremia. The functional test for the kidneys that is being most generally employed by both internists and surgeons in America is that of phenolsulphonephthalein. In many cases of uremia the results secured by this test accord with the clinical data secured by other procedures, but there are notable exceptions in the operation of the test.

The author cites three cases representing three different types of nephritis, in which the diagnosis was made correctly in each instance and was proved by necropsy. The phenolsulphonephthalein test gave a figure approximating normal in all, yet as each case terminated fatally, one would not assert that the renal function was normal. As to prognosis, this test would have indicated that his recovery was almost certain. These cases suggest that the excretion of phenolsulphonephthalein depends on some other factor (circulation?) than pure renal disease, which by its presence or absence determines the rate of secretion by the kidney, probably non-protein nitrogen of the blood.

In the last few years there is notable in the literature an ever increasing insistence on the significance of the non-protein nitrogen (residual, filtrate, incoagulable nitrogen) of the blood in the study of nephritis. When the non-protein nitrogen is so large, as in some cases up to 200 milligrams per 100 cubic centimeters of blood, the outcome of the patient is pretty definitely bad. But these figures even in uremia are not the rule, and it is not at all unusual to find the non-protein nitrogen within the normal variation in cases of chronic interstitial nephritis with high blood pressure. The author cites a case of severe nephritis, with retinal changes, cardiac hypertrophy, and albumin and casts in the urine, with non-protein nitrogen 28 milligrams in 100 cubic centimeters of blood. The clinical diagnosis in this case was uremia, and death occurred on the fifth day after admission.

The author concludes that such tests and methods of investigation are undoubtedly valuable. Correctly applied they stimulate interest and lead in the long run to broadened knowledge. But just so soon as such methods are regarded as ultimate criteria, we are led first into errors in diagnosis, and from that creep into narrowed medicine and false ideas as to the pathology of a disease entity.

